# SARS-CoV-2 Seropositivity among US Marine Recruits Attending Basic Training, United States, Spring–Fall 2020

**DOI:** 10.3201/eid2704.204732

**Published:** 2021-04

**Authors:** Andrew G. Letizia, Yongchao Ge, Carl W. Goforth, Dawn L. Weir, Rhonda Lizewski, Stephen Lizewski, Alessandra Soares-Schanoski, Sindhu Vangeti, Nada Marjanovic, Stuart C. Sealfon, Irene Ramos

**Affiliations:** Naval Medical Research Center, Silver Spring, Maryland, USA (A.G. Letizia, C.W. Goforth, D.L. Weir);; Icahn School of Medicine at Mount Sinai, New York, New York, USA (Y. Ge, A. Soares-Schanoski, S. Vangeti, N. Marjanovic, S.C. Sealfon, I. Ramos);; Naval Medical Research Unit 6, Lima, Peru (R. Lizewski, S. Lizewski)

**Keywords:** SARS-CoV-2, COVID-19, seroprevalence, military recruits, young adults, coronavirus disease, severe acute respiratory syndrome coronavirus 2, viruses, respiratory infections, zoonoses, United States

## Abstract

In a study of US Marine recruits, seroprevalence of severe acute respiratory syndrome coronavirus 2 IgG was 9.0%. Hispanic and non-Hispanic Black participants and participants from states affected earlier in the pandemic had higher seropositivity rates. These results suggest the need for targeted public health strategies among young adults at increased risk for infection.

Coronavirus disease (COVID-19) cases are increasing in young adults (*1*). In some instances, prevalence among younger adults exceeds that of older adults (*2*). Younger adults often have a paucisymptomatic or asymptomatic response to infection (*3*). The potential for rapid spread exists within this age group (*4*). Without active serologic surveillance, cases among young adults might not be identified and the cumulative incidence underestimated. Well-defined cohorts are needed to assess the proportion of young adults who have severe acute respiratory syndrome coronavirus 2 (SARS-CoV-2) antibodies (*5*). We studied the seroprevalence of SARS-CoV-2 IgG among US Marine recruits preparing for basic training at Marine Corps Recruit Depot Parris Island, South Carolina.

## The Study

Before beginning basic training, recruits quarantined for 2 weeks at a hotel or college campus as previously described (*6*). Within 48 hours of arriving at the quarantine location, ≈350–500 recruits per week were offered the opportunity to volunteer for the COVID-19 Health Action Response for Marines Study, which included collecting baseline SARS-CoV-2 serologic test results.

We collected paper questionnaires and assayed serum samples for the presence of SARS-CoV-2 IgG upon participants’ arrival at the quarantine location. We tested serum specimens for SARS-CoV-2 IgG by ELISA (*6*) (Appendix). The association between demographics, risk factors, and IgG-positivity variables were analyzed with logistic regression to determine the p value and odds ratio (OR). 

The study protocol was approved by the Naval Medical Research Center Institutional Review Board in compliance with all applicable Federal regulations governing the protection of human subjects. All participants provided written informed consent for participation.

**Figure 1:**
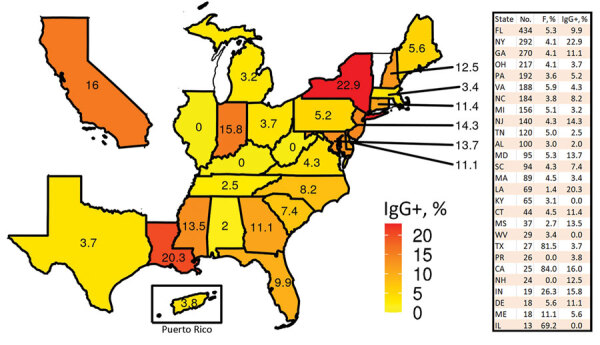
Percentage of severe acute respiratory syndrome coronavirus 2 IgG-positive recruits from US states or territories with >10 participants in the COVID-19 Health Action Response for Marines Study, May 11–September 7, 2020. The table lists the number of participants and the percentage of women. .

During May 11–September 7, 2020, we enrolled 3,249 (69.8%) volunteers out of 4,657 eligible recruits; because the minimum age was 18, 530/5,187 (10.2%) persons who were 17 years of age were ineligible. Valid IgG data were obtained for 3,196/3,249 (98.4%) participants. Most participants were from the Eastern United States or states with larger populations (Figure 1). Study participants had a median age of 19.1 (range 18–31) years, and 257 (8.0%) were women (Table 1). Participants 18–20 years of age (2,748 [86.0%]) were overrepresented in our cohort compared with 3.9% in the general US population according to 2020 Census data. When compared with 2020 Census data for persons 18–20 years of age, our cohort had a similar percentage of Hispanic participants (23.9% compared with 23.9%) and non-Hispanic Black participants (12.04% compared with 15.04%) (*7*).

**Table 1:** Demographics of 3,196 COVID-19 Health Action Response for Marines study participants with valid IgG data, United States, May 11–September 7, 2020*

Characteristic	Total
Age, y, mean (SD)	19.1 (1.9)
Sex	
M	2,939 (92.0)
F	257 (8.0)
Race or ethnicity	
Non-Hispanic White	1,817 (56.9)
Non-Hispanic Black	414 (13.0)
Non-Hispanic other†	197 (6.2)
Hispanic	768 (24.0)
IgG	
Negative	2,907 (91.0)
Positive	289 (9.0)
COVID-19 by PCR	
Negative	3,054 (95.6)
Positive	28 (0.9)
Other‡	114 (3.6)
State group§	
Early spring	701 (21.9)
Late spring	1,389 (43.5)
Summer	994 (31.1)
Other‡	112 (3.5)
Resides in a country other than the United States	
No	3,084 (96.5)
Yes	23 (0.7)
Other‡	89 (2.8)
Born in a country other than the United States	
No	2,928 (91.6)
Yes	231 (7.2)
Other‡	37 (1.2)

**Figure 2:**
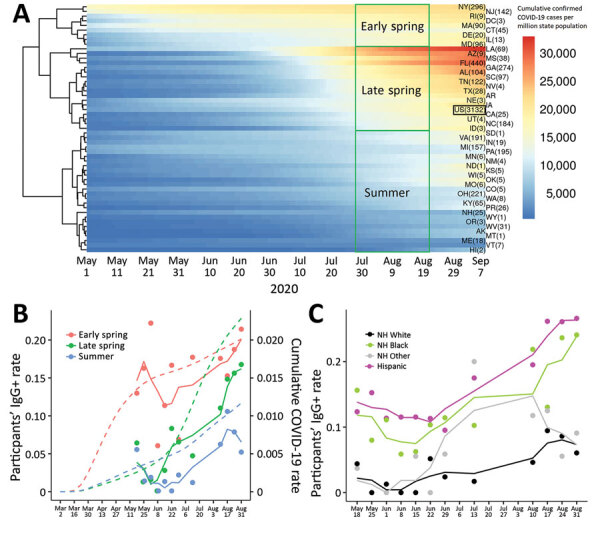
Confirmed COVID-19 and severe acute respiratory syndrome coronavirus 2 (SARS-CoV-2) IgG-positivity by state, race, and ethnicity, in a study of US Marine recruits, May 11–September 7, 2020. A) Heatmap of cumulated confirmed COVID-19 cases normalized by each state’s population. Each row represents 1 state, and number in parentheses indicates number of participants. Color reflects cumulative PCR-confirmed cases per 1 million state population (data obtained from COVID-19 Data Repository by the Center for Systems Science and Engineering at Johns Hopkins University, https://github.com/CSSEGISandData/COVID-19). Each column indicates 1 day during May 1–September 7, 2020. The US in aggregate is shown in the black box. B) SARS-CoV-2 IgG-seropositivity rate by week of enrollment on the basis of state groupings. Colored dots indicate the weekly IgG-positivity rate for study participants grouped by state; colored solid lines show 3-week running means. Dotted lines indicate cumulative PCR-confirmed COVID-19 cases in each state grouping obtained from COVID-19 Data Repository, including data before the study commenced. C) SARS-CoV-2 IgG-positivity by race and ethnicity. Colored dots indicate weekly IgG-positivity rate for study participants; colored solid lines indicate 3-week running means. Because of the relatively small number of participants in the first study week (May 11), they are merged into May 18 data. COVID-19, coronavirus disease; NH, Non-Hispanic.

*Values are no. (%) except as indicated. COVID-19, coronavirus disease.  †Non-Hispanic other also includes participants with missing values. ‡Inconclusive assay or the participant left question blank or answered by marking unknown.  §Defined in Figure 2, panel A, and the Appendix (https://wwwnc.cdc.gov/EID/article/27/4/20-4732-App1.pdf).

Upon arrival at quarantine, 28/3,196 (0.9%) participants were SARS-CoV-2–positive by PCR and 289/3,196 (9.0%) were ELISA-positive for SARS-CoV-2 IgG targeting the receptor-binding domain of the spike protein. A total of 135/768 (17.6%) participants who identified as Hispanic were positive for SARS-CoV-2 IgG (Table 2), higher than the percentage of non-Hispanic White participants (80/1,817 [4.4%]) (OR 3.80, 95% CI 2.82–5.14; p<0.001). Hispanic participants also had higher rates of IgG seropositivity among weekly cohorts throughout the study period, and those rates increased with time (trend p<0.00017); seropositivity rates rose from 12.1% in May and June to 22.3% in July and August. Similarly, non-Hispanic Black participants had higher prevalence of SARS-CoV-2 IgG (62/414 [15.0%]) than non-Hispanic White participants (OR 3.54, 95% CI 2.47–5.05; p<0.001). Seropositivity was also greater in women (32/257, 12.5%) than men (257/2,939, 8.7%) (OR 1.57, 95% CI 1.02–2.33; p = 0.033).

**Table 2:** Association between demographic variables and SARS-CoV-2 IgG results in study of seroprevalence in US Marine recruits, United States, May 11–September 7, 2020*

Characteristic	IgG result			Univariable analysis			Multivariable analysis	
	Negative	Positive		OR (95% CI)	p value		OR (95% CI)	p value
Age, y, mean (SD)	19.1 (1.9)	19.0 (1.7)		0.99 (0.92–1.05)	0.672		0.96 (0.89–1.02)	0.212
Sex								
M	2682 (91.3)	257 (8.7)		Referent			Referent	
F	225 (87.5)	32 (12.5)		1.48 (0.99–2.17)	0.048		1.57 (1.02–2.33)	0.033
Race or ethnicity								
Non-Hispanic White	1737 (95.6)	80 (4.4)		Referent			Referent	
Non-Hispanic Black	352 (85.0)	62 (15.0)		3.82 (2.69–5.42)	<0.001		3.54 (2.47–5.05)	<0.001
Non-Hispanic Other	185 (93.9)	12 (6.1)		1.41 (0.72–2.54)	0.283		1.32 (0.67–2.39)	0.388
Hispanic	633 (82.4)	135 (17.6)		4.63 (3.47–6.22)	0.001		3.80 (2.82–5.14)	<0.001
State group								
Early spring	591 (84.3)	110 (15.7)		Referent			Referent	
Late spring	1263 (90.9)	126 (9.1)		0.54 (0.41–0.71)	<0.001		0.61 (0.46–0.81)	0.001
Summer	951 (95.7)	43 (4.3)		0.24 (0.17–0.35)	<0.001		0.35 (0.23–0.50)	<0.001
Other†	102 (91.1)	10 (8.9)		0.53 (0.25–0.99)	0.065		0.54 (0.25–1.04)	0.085

*Values are no. (%) except as indicated. Univariable odds ratio and p value were computed on the basis of the logistic regression with a single variable only. Multivariable odds ratio and p value were computed by using all 4 variables in the model. OR, odds ratio; SARS-CoV-2, severe acute respiratory syndrome coronavirus 2. †Inconclusive assay (not residing in the United States) or participants left residence question blank or answered by marking unknown.

Because participants came from states that were affected by COVID-19 at different times and in variable intensity, we grouped participants’ states of origin into 3 categories on the basis of when confirmed COVID-19 cases began to increase in each state (Appendix) (*8*). The groups were early spring, for states in which the outbreak began in March; late spring, for states in which the outbreak began in early June; and summer, for states in which the outbreak began in late June–July (Figure 2, panel A). We plotted weekly IgG-positivity rates during the 17-week study period (Figure 2, panel B) and found that participants from the early spring states had higher IgG seropositivity compared with late spring and summer and maintained a similar rate for the duration of the study. Overall, SARS-CoV-2 IgG seropositivity among participants from summer states (43/994 [4.3%]) and late spring states (126/1,389 [9.1%]) was much lower than in participants from early spring states (110/701 [15.7%]); OR was 0.35 (0.23–0.50; p<0.001) for summer and late spring states and 0.61 (0.46–0.81; p = 0.001) for early spring states. Figure 2, panel C, shows the weekly IgG-positive rate by race and ethnicity.

## Conclusions

By using a cross-sectional study design during a 17-week period, the baseline seroprevalence of IgG against SARS-CoV-2 in US Marine recruits primarily from the eastern United States was 9.0%. In the United States, young adults have demonstrated higher levels of SARS-CoV-2–specific antibodies than persons of other ages (*9*). Among persons 18–20 years of age, low adherence to recommendations for social distancing, wearing of masks, and other public health measures might increase their level of exposure compared with older persons (*10*). The high rate of asymptomatic infection in this age group (*6*) likely leads to underestimates of the cumulative incidence. Subsequent spread could contribute to infections among more vulnerable populations (*11*). Therefore, this age group represents an at-risk population that should be considered for COVID-19 monitoring and other targeted public health measures.

The participants in our study did not come from a cohort of convenience, a group at high risk, or a group receiving medical care; rather, they were selected from a group of young adults for the primary purpose of assessing baseline seropositivity. This process minimized selection bias (*12*), excluding the self-selection that occurred because participants chose to join the US Marine Corps and enroll in our study. Enrollment rate (70%) was high, which increased the likelihood that we studied a representative sample of recruits.

Consistent with other reports (*13*), Hispanic participants had higher IgG seroprevalence (OR 3.80) than non-Hispanic White participants in a multivariable logistic regression. This trend was similar for non-Hispanic Black participants and participants residing in states affected earlier in the pandemic. Our cohort was primarily young adults, many of whom had never held full-time jobs and might not represent essential workers, who have been associated with higher rates of infection among minority groups (*14*). It has been proposed that the higher incidence of SARS-CoV-2 in minority communities is associated with lower socioeconomic status and the associated inability to telecommute, leading to increased workplace exposure (C.T. Rentsch, unpub. data, https://doi.org/10.1101/2020.05.12.20099135). Instead, these data could demonstrate the downstream effects of residing with an essential worker or could reflect intrinsic risk within a community.

Our study incorporates participants from multiple states and represents a diverse mix of race, ethnicity, and backgrounds, providing a unique assessment relevant to public health concerns among persons 18–20 years of age. Conversely, the study results are not representative of the population as a whole, especially children and older adults. Even among young adults, the results are specific to persons who chose to join the US Marine Corps. Additional limitations include a lack of information regarding exposure, participant risk-taking behavior before enrollment, and lack of confirmation of COVID-19 by PCR before study enrollment.

In our study, the seroprevalence of SARS CoV-2 IgG among a cohort of predominately young men was 9.0%. Multivariable analysis showed incidence rates were significantly higher in women, Hispanic participants, Non-Hispanic Black participants, and participants from states that were affected earlier in the pandemic. These data can help inform surveillance and management strategies, as well as targeted public health interventions, for this age group.

Appendix Additional information about SARS-CoV-2 seropositivity among US Marine recruits attending basic training, United States, spring–fall 2020.
