# Supplementary material for: SARS-CoV-2 Seropositivity among US Marine Recruits Attending Basic Training, United States, Spring–Fall 2020
Source: Emerg Infect Dis. 2021 Apr;27(4):1188–92. doi: 10.3201/eid2704.204732 (PMC8007328; doi:10.3201/eid2704.204732)
Supplement: Appendix — Additional information about SARS-CoV-2 seropositivity among US Marine recruits attending basic training, United States, spring–fall 2020. [file 20-4732-Techapp-s1.pdf]

Article DOI: https://doi.org/10.3201/eid2704.204732

SARS-CoV-2 Seropositivity among US Marine Recruits Attending Basic Training, United States, Spring–Fall 2020

Appendix

Methods

Study Design and Participants

After recruits chose to join the United States Marine Corps, they attended basic training at 1 of 2 locations: Marine Corps Recruit Depot San Diego in California or Marine Corps Recruit Depot Parris Island (MCRDPI) in South Carolina. The location a recruit attended was primarily determined by the geography of the recruit's state of residence; states East of the Mississippi River, in general, go to MCRDPI and those to the West attend San Diego. Exceptions were made for administrative reasons with regard to recruit training class size. However, all female recruits attended MCRDPI. These procedures help to explain the large proportion of study participants from the Eastern United States and the high prevalence of women from Western states that have larger populations.

Once a recruit was assigned a training date and location, they were instructed to quarantine at home for 14 days. A recruiter, wearing a mask and maintaining maximum possible distance, would transport the recruit, who was also masked, in a vehicle to a local Military Entrance Processing Station where a provider performed a history and physical examination on the recruit. If deemed physically and mentally fit for Marine Corps enlistment, the recruit traveled by bus or plane to the quarantine campus or hotel. Recruits were instructed to wear masks at all times and maintain social distancing of $\geq$6 feet and avoid interactions with others while traveling. Once a recruit arrived at the local airport or bus station, they were picked up by van or bus and transported to the supervised quarantine location, where they observed the same COVID-19 mitigation strategies for an additional 14 days. The quarantine settings were selected for the specific purpose of strictly enforced public health measures implemented for the entire 2

weeks. The recruits and staff were forbidden to leave and no visitors, other than persons delivering supplies and food, local essential workers, and study staff, were allowed onto the premises. All of these measures were enforced by Marines at all times. Specific public health measures have been previously described (*1*).

Within 48 hours of arriving at the quarantine location, ≈350–500 recruits per week were offered the opportunity to volunteer for the COVID-19 Health Action Response for Marines (CHARM) Study, which included collecting baseline SARS-CoV-2 serologic test results. Recruits were eligible if they were ≥18 years and could complete follow-up encounters. Recruits 17 years of age were ineligible. Study enrollment occurred after recruits had been in-processed and had personal effects (including cell phones) secured, rooms assigned, and gear issued. The recruits attended a group consent brief of 50–100 participants using an ombudsman who explained the study, exactly what was being asked of participants, risks, benefits, and the state of COVID-19 in the recruit setting. Since recruits are a vulnerable population and at risk for coercion, special measures were undertaken including study briefers, who are active duty Navy personnel, wearing civilian clothes, not disclosing military ranks, not having members in the recruit's chain of command present, and ensuring that participation would not affect a recruit's medical care or influence the grading of a recruit's military performance.

Institutional Review Board approval was obtained from the Naval Medical Research Center (protocol no. NMRC.2020.0006) in compliance with all applicable federal regulations governing the protection of human subjects. All participants provided written informed consent for study participation.

Procedures

Recruits consented to undergo a mid-turbinate nares swab for SARS-CoV-2 qPCR testing and blood draw upon enrollment that included serum. We collected paper questionnaires (Appendix Figure) to identify demographics, risk factors, and symptoms, and assayed serum for the presence of SARS-CoV-2 IgG upon arrival at the quarantine location. Data was first recorded in Microsoft Excel spreadsheets before automated integration with the statistical programming language R 3.6.3 (*2*). The data collected included sex, age, ethnicity, race, place of birth, state or country of residence, medical history including smoking or vaping or exposure to secondhand smoke, and risk factors including use of masks, practicing self-quarantine before arrival, recent

travel, known exposure to persons with COVID-19, and exposure to someone with flu-like or other respiratory illness.

Laboratory Methodology

Presence of SARS-CoV2 IgG in serum was evaluated using ELISA with some modifications from Amanat et al. (*3*), as previously described (*1*). Briefly, 384-well Immulon 4 HBX (Thermofisher, https://www.thermofisher.com) plates were coated overnight at 4°C with recombinant His-tagged Spike (S) receptor-binding domain (RBD) (SinoBiological, https://www.sinobiological.com) at a concentration of 2 µg/ml in phosphate-buffered saline (PBS). Plates were washed 3 times with 0.1% Tween-20 (Fisher Scientific) PBS (PBS-T) using an automated ELISA plate washer (Aquamax 4000, Molecular devices), and blocked for 1 h at room temperature (RT) with 3% milk PBS-T. Blocking solution was removed, and serum samples diluted in 1% milk PBS-T were dispensed in the wells. At least 2 positive controls (serum samples with known SARS-CoV-2 IgG presence), 8 negative controls (serum samples collected before July 2019) and 4 blanks (no serum) were included in every plate. Plates were incubated for 2 h at room temperature and washed 3 times with PBT-T. Next, peroxidase conjugated goat F(ab')2 Anti-Human IgG (abcam) were added at a dilution 1:5,000–1:10,000 dilutions (determined after optimization for each antibody lot) in 1% milk PBS-T, and plates were incubated for 1 h at RT. Plates were washed 6 times with PBS-T, developed by using SIGMAFAST OPD (Sigma-Aldrich, https://www.sigmaaldrich.com), and the reaction was stopped after 10 min with 3M HCl. Optical density (OD) at 492 nm was measured by using a Spectramax M2 microplate reader (Molecular Devices, https://www.moleculardevices.com). All serum samples were screened at a 1:50 dilution. Those samples with an OD 492 nm value higher than the average of the negative controls plus 3 times their SD in the screening underwent titration assay (6 serial 1:3 serum dilutions starting at 1:50). Serum samples were considered positive when at least 2 consecutive dilutions showed higher OD 492 nm than the average of the negative controls plus 3 times their SD at the correspondent dilution or 0.15 OD 492 nM.

Statistical Analyses

Analyses, figures, and tables were generated by using R 3.6.3 (*2*). Associations between demographics, risk factors, and IgG-positivity variables were analyzed with logistic regression to compute the p value and the odds ratio. None of the risk factor data (Appendix Figure) was statistically significant and is not displayed. Significance was a priori established at <0.05.

The logistic regression is analyzed with 2 approaches: a) single variable approach: $\log \frac{p}{1-p} = \beta_0 + \beta x$ and b) multivariate approach: $\log \frac{p}{1-p} = \beta_0 + \beta_1 x_1 + \beta_2 x_2 + \cdots + \beta_p x_p$. Note that when variable x_i is a categorical variable with L_i possible values coded from 1 to L_i and the code 1 is for the reference group, $\beta_i x_i$ should be understood as $\sum_{j=2}^{L_i} I(x_i = j)\beta_{i,j}$.

The collinearity for the variables in the multivariable logistic regression was assessed by using GVIF (generalized variance-inflation factors) (*4*). $GVIF^{\frac{1}{2*df}}$ (df is the degree of freedom of the variable) is computed for all variables in this paper. All variables were less than 1.06, indicating collinearity did not impact the analysis or violate assumptions. The collinearity is also assessed by the conditioner number which is ≈12, less than the 30, also indicating weak collinearity.

The trend test for the weekly IgG-positive rate of participants of Hispanic ethnicity is based on the Cochran-Armitage test. Because of the relatively small number of participants in the first study week (May 11), the participants' weekly IgG-positive rates have been smoothed with a 3-week running mean.

Race and ethnicity were categorized as non-Hispanic White, non-Hispanic Black, non-Hispanic Other, and Hispanic. A total of 18/3,196 (0.6%) participants did not supply any information on race or ethnicity and were grouped into the non-Hispanic Other category.

The 2020 US census data was downloaded from https://www.census.gov/data/tables/2020/demo/popest/2020-demographic-analysis-tables.html on December 20, 2020. The data contain information regarding the percentage of the US population that identifies as Black or Hispanic for each age year, but subcategories of race for the non-Hispanic population are still unavailable. For this reason, we compared data for the Black category, which was available in the census data, with data for non-Hispanic Black participants within our study. Specifically, we focused on the percentage of Black and Hispanic persons 18–20 years of age in the general population compared with our non-Hispanic Black and Hispanic study participants within the same age range.

Daily COVID-19 cases confirmed by viral tests during January 22–September 7 were downloaded from the COVID-19 Data Repository of the Center for Systems Science and Engineering (CSSE) at Johns Hopkins University (*5*). The heatmap in Figure 2, panel A

(https://wwwnc.cdc.gov/EID/article/27/4/20-4732-F2) represents the cumulative confirmed COVID-19 cases per 1 million of the state population. In the heatmap, states are clustered by temporary profiles of cumulative confirmed COVID-19 cases, as indicated by the dendrogram, which separates the states into 3 major groups. The groups were Early Spring, for states in which the first outbreak began in March; Late Spring, for states in which the outbreak began in early June; and Summer, for states in which the outbreak began in late June–July. The overall profile of the whole country (labeled US on 1 row) is in the Late Spring group and is placed in a black box. We used the aggregated data of each state group to compute the cumulated rate (dotted lines in Figure 2, panel B, right axis); the first outbreak is identified by the first local maximum slope. Our study had 701 (21.9%) participants from Early Spring states, 1,389 (43.5%) from Late Spring states, and 994 (31.1%) from Summer states. A total of 112 (3.5%) participants were not included in the analysis since they resided in a foreign country or did not provide a residence.

Specificity and Sensitivity of SARS-CoV-2 S-RBD IgG Serologic Test

To determine the specificity of the S-RBD IgG ELISA assay, we used 70 commercial serum samples drawn before July 2019 (44 purchased from BioChemed Services and 26 provided by Dr. Russell Tracy, Larner College of Medicine, University of Vermont, Burlington, Vermont, USA). To determine the sensitivity of the assay, we used 51 serum samples from subjects that had been previously confirmed as SARS-CoV-2–positive by PCR ≥14 days before serum sample collection (all of them were <90 days from PCR-positive test). All samples were screened at a 1:50 dilution, and those identified as positive were titered using 6 serial 1:3 serum dilutions (starting at 1:50). Those with at least 2 positive consecutive dilutions in the titration step (titer of 1:150) were considered seropositive. This assay was shown to have a 97.14% specificity (95% CI 93.24–100.00) and 96.08 sensitivity (95% CI 90.75–100.00).

References

<jrn>1. Letizia AG, Ramos I, Obla A, Goforth C, Weir DL, Ge Y, et al. SARS-CoV-2 Transmission among Marine recruits during quarantine. N Engl J Med. 2020;383:2407–16. https://doi.org/10.1056/NEJMoa2029717 PubMed</jrn>

<eref>2. R Project for Statistical Computing. R: A language and environment for statistical computing. 2020 [cited 2020 Feb 29]. http://www.R-project.org/</eref>

<jrn>3. Amanat F, Stadlbauer D, Strohmeier S, Nguyen THO, Chromikova V, McMahon M, et al. A serological assay to detect SARS-CoV-2 seroconversion in humans. Nat Med. 2020;26:1033–6. https://doi.org/10.1038/s41591-020-0913-5 PubMed</jrn>

<jrn>4. Fox J, Monette G. Generalized collinearity diagnostics. J Am Stat Assoc. 1992;87:178–83. https://doi.org/10.1080/01621459.1992.10475190</jrn>

<jrn>5. Dong E, Du H, Gardner L. An interactive web-based dashboard to track COVID-19 in real time. Lancet Infect Dis. 2020;20:533–4. https://doi.org/10.1016/S1473-3099(20)30120-1 PubMed</jrn>

Initial Questionnaire Version 1 14APRIL2020
COVID-19 Health Action Response for Marines (CHARM) Study

Completed by Study Personnel

Participant ID: PI-_____-_______ Visit Number _______________

Sample ID (PI-XXXX): PI-____________ Samples Collected: ☐ Sputum ☐ Nares Swab ☐ Blood

(PI=Parris Island; Initials=First Middle Last; XXXX= sample number)

Date: ____/____/______ Name: ____ SSN Last 4:_______

Last, First, Middle Initial

Phone Number:____________________ Email:______________________________________

Drill Instructor Name:______________________________ Company/Platoon:_____________ /_____________

Demographics

Age:_____(years) Sex: ☐ Male ☐ Female Ethnicity: ☐ Hispanic/Latino ☐ Non-Hispanic/Latino ☐ Not specified

Race: ☐ Asian ☐ American Indian/Alaska Native ☐ Black ☐ Native Hawaiian/Other Pacific Islander ☐ White ☐ Other, specify:______________________

Birthplace:_______________________ Region of primary residence:___________________________

Have you ever resided outside of the US for greater than 1 month? ☐ Yes ☐ No

If yes, specify:____

Marine Corps Recruit Depot, Parris Island (PI) Information

Date arrived:___/___/______ Recruit class:____________

Have you been exposed to anyone with flu-like illness since arriving? ☐ Yes ☐ No ☐ Unk

If yes, specifiy when and where:____

List all locations you visited 14 days prior to arrival:____

____

Did you practice self-quarantine or isolation at home prior to arrival? ☐ Yes ☐ No

Barracks location: ______________________________

How far away do you sleep from someone else? ☐ <6 feet ☐ >6 feet ☐ No one else in the room

What type of personal protection are you using? (check all that apply)

☐ None ☐ Surgical mask ☐ Cloth mask ☐ Other, specify:_______________________________

Pre-existing medical conditions?

History of asthma	☐ Yes	☐ No	
Current smoker (including vaping)	☐ Yes	☐ No	If yes, for how many years: _______
Former smoker (including vaping)	☐ Yes	☐ No	If yes, date quit:___/___/_____
Family smoking history/second-hand exposure	☐ Yes	☐ No	If yes, specify:___________________

Other, specify: ____

NAVY MEDICINE HRPP
HRPP#: NMRC . 2020 . 0006
Approval Date: 03 May 2020
Expiration Date: 30 April 2021
Verified By: TKS

Initial Questionnaire Version 1 14APRIL2020

COVID-19 Health Action Response for Marines (CHARM) Study

Within the last 14 days have you had any of the following exposures (check all that apply):

- ☐ Travel to mainland China or other non-US country Specify:____________________
- ☐ Known contact with a lab-confirmed COVID-19 case-patient?
- ☐ Hospital, clinic, or other medical facility
- ☐ Other, specify:___________________________________
- ☐ Exposure to anyone with severe acute lower respiratory distress (difficulty breathing)?
- ☐ Exposure to anyone with flu-like symptoms?
- ☐ Contact with animals. Specify: ____________
- ☐ Unknown

Have you experienced any of the following flu-like symptoms within the last 14 days?

Fever >100.4F (38C)	☐ Yes	☐ No	☐ Unk
Subjective fever (felt feverish)	☐ Yes	☐ No	☐ Unk
Chills	☐ Yes	☐ No	☐ Unk
Muscle aches	☐ Yes	☐ No	☐ Unk
Fatigue	☐ Yes	☐ No	☐ Unk
Runny nose	☐ Yes	☐ No	☐ Unk
Sore throat	☐ Yes	☐ No	☐ Unk
Cough (new onset or worsening of chronic cough)	☐ Yes	☐ No	☐ Unk
Shortness of breath	☐ Yes	☐ No	☐ Unk
Nausea or vomiting	☐ Yes	☐ No	☐ Unk
Headache	☐ Yes	☐ No	☐ Unk
Decreased ability to taste or smell	☐ Yes	☐ No	☐ Unk
Abdominal pain	☐ Yes	☐ No	☐ Unk
Diarrhea (≥3 loose/looser than normal stools/24hr period)	☐ Yes	☐ No	☐ Unk

Other, specify: ____

Page 2

NAVY MEDICINE HRPP
HRPP#: NMRC . 2020 . 0006
Approval Date: 03 May 2020
Expiration Date: 30 April 2021
Verified By: TRB

Adapted from CDC PUI Screening Tool Page 1

Appendix Figure. Questionnaire administered to participants in COVID-19 Health Action Response for Marines study, May 11–September 7, 2020.
